# A Digital Health Weight Loss Program in 250,000 Individuals

**DOI:** 10.1155/2020/9497164

**Published:** 2020-03-26

**Authors:** Conor Senecal, Robert Jay Widmer, Beth R. Larrabee, Mariza de Andrade, Lilach O. Lerman, Amir Lerman, Francisco Lopez-Jimenez

**Affiliations:** ^1^Department of Cardiovascular Medicine, Mayo Clinic, Rochester, MN, USA; ^2^Department of Cardiology, Baylor College of Medicine, Houston, TX, USA; ^3^Division of Biostatistics, Mayo Clinic College of Medicine, Rochester, MN, USA; ^4^Division of Nephrology and Hypertension, Department of Internal Medicine, Mayo Clinic, Rochester, MN, USA

## Abstract

**Objective:**

To evaluate whether individuals following a weight loss program based on a mobile application, wireless scale, and nutritional program but no face-to-face care can achieve clinically significant weight loss in a large cohort.

**Design:**

Retrospective observational analysis. *Setting*. China from October 2016 to December 2017. *Participants*. Mobile application users with a minimum of 2 weights (baseline and ≥35 days). *Intervention*. A commercial (Weijian Technologies) weight loss program consisting of a dietary replacement, self-monitoring using a wireless home scale, and frequent guidance via mobile application. *Main Outcome*. Mean weight change around 42, 60, 90, and 120 days after program initiation with subgroup analysis by gender, age, and frequency of use.

**Results:**

251,718 individuals, with a mean age of 37.3 years (SD: 9.86) (79% female), were included with a mean weight loss of 4.3 kg (CI: ±0.02) and a mean follow-up of 120 days (SD: 76.8 days). Mean weight loss at 42, 60, 90, and 120 d was 4.1 kg (CI: ±0.02), 4.9 kg (CI: ±0.02), 5.6 kg (CI: ±0.03), and 5.4 kg (CI: ±0.04), respectively. At 120 d, 62.7% of participants had lost at least 5% of their initial weight. Both genders and all usage frequency tertiles showed statistically significant weight loss from baseline at each interval (*P* < 0.001), and this loss was greater in men than in women (120 d: 6.5 vs. 5.2 kg; *P* < 0.001). The frequency of recording (categorized as high-, medium-, or low-frequency users) was associated with greater weight loss when comparing high, medium, and low tertile use groups at all time intervals investigated (e.g., 120 d: −8.6, −5.6, and −2.2 kg, respectively; *P* < 0.001).

**Conclusions:**

People following a commercially available hybrid weight loss program using a mobile application, wireless scale, and nutritional program without face-to-face interaction on average achieved clinically significant short- and midterm weight loss. These results support the implementation of comparable technologies for weight control in a large population.

## 1. Introduction

Obesity is a worldwide epidemic that continues to grow in scale [1]. It is closely related to many disease states, including cardiovascular disease, diabetes, and several forms of malignancy [2]. Even moderate weight loss (5% of body weight) can translate to meaningful disease prevention [3]. Effective lifestyle programs have been developed to combat obesity; however, these programs are difficult to scale and are costly and location-specific [4]. Digital health weight loss programs have attractive features of easy dissemination, relatively low cost, and scalability [5]. If these tools are able to effectively facilitate weight loss in individuals, they could be scaled to help curb the obesity epidemic that has coincided with a dramatic rise in smartphone use and Internet connectivity [6].

A number of approaches using mobile and web-based applications alone or in conjunction with food supplements or replacements have been studied with mixed results [7]. While the majority of studies have shown some evidence of weight loss, they were limited by small sample size and heterogeneity of methods [5, 7]. Specific interventions such as smartphone-based applications [8, 9] and wireless scales [10] seem to be particularly effective. Further research into specific interventions in large and diverse populations and over extended time periods would further clarify the role of these tools in obesity treatment [11].

Adherence to weight loss programs has previously been shown to be an important predictor of success [12]. Small studies have shown increased adherence with the use of mobile application as opposed to paper-based methods for weight monitoring [13]. However, the association between mobile application adherence and weight loss requires further exploration [8].

The aim of this study was to evaluate the use of digital health platform for weight loss utilizing a mobile application and wireless scale in a large retrospective observational study with attention to differences among demographics and frequency of use.

## 2. Methods

### 2.1. MetaWell

**Figure 1:**
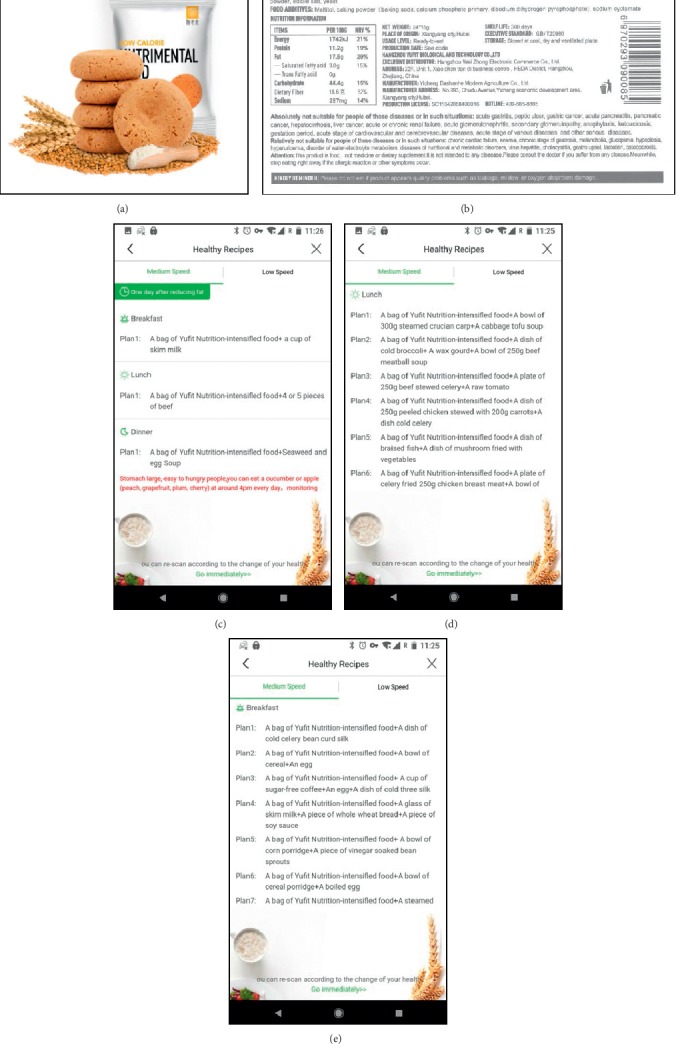
Nutritional Program. (a) Meal replacement biscuit. (b) Nutritional information provided on meal replacement packaging. (c) Daily summary of nutritional plan. (d) Lunch meal options. (e) Breakfast meal options.

**Figure 2:**
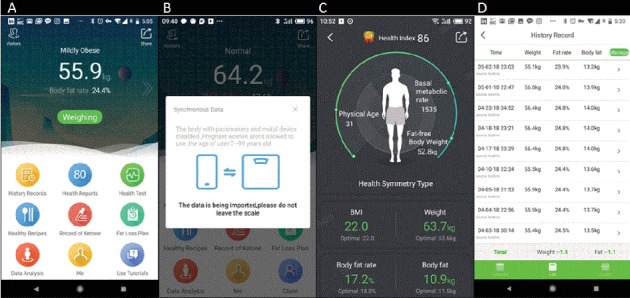
Screenshots of the MetaWell mobile application. (a) Home screen of the application. (b) Weighing screen, shown as users are on scale. (c) Health summary screen. (d) Record of weight change.

The MetaWell program (Weijian Technologies Inc., Hangzhou, China) is an entirely remote weight loss program, without face-to-face interaction, that consists of a free mobile application combined with a wireless home scale and nutrition program. The MetaWell application is available in the Google Play and Apple App stores. Upon download, users are prompted to register and provide basic demographic information including age and gender. They are also prompted to purchase the associated wireless scale ($45), a device that captures body weight and bioimpedance measurements, including water content and fat percent. This investigation was focused on weight change only. The program focuses predominantly on weight loss via diet by providing a low-calorie meal plan centered around meal replacement biscuits supplemented with healthy recipes available in the application. While engaged in the program, participants receive individualized meal plans from the application recommending up to 3 Yufit biscuits daily along with a selection of other recommended foods with the aim of negative caloric balance. The program is tailored based on each participant's basal metabolic requirements. Yufit biscuits are low-calorie, noncaffeinated meal replacements with a low glycemic index. They are available for retail purchase as a part of the weight loss program. Their macronutrient profile is 416 calories per 100 g, with 11.2 g of protein, 17.5 g of fat, and 44.4 g of carbohydrates. Complete nutritional information and sample diet plans provided in the app are detailed in Figure 1. The biscuits have not previously been studied. Direct measures of supplement use during the period studied were unavailable. Activity is encouraged during the program, but no specific exercise program is provided. Users are prompted by the application to weigh themselves on a daily basis via the wireless scale. In the application, participants can see a record of their weight loss progress, as well as a “Health Status Overview” that provides a snapshot of their current health data and optimal measures using a goal BMI of 22.0 kg/m^2^. Screen captures of the application are presented in Figure 2. If at the initiation of the program or throughout the course of the program a person body weight reaches a BMI of <22.0 kg/m^2^, the program directs them to maintain weight as opposed to continue weight loss.

### 2.2. Study Protocol

Using a retrospective design, we investigated the data to determine if active MetaWell users on average achieved weight loss and if any recorded factors were associated with more or less weight loss among program's users. A complete, deidentified record of application users collected by Weijian Technologies Inc. was provided for research purposes. Participants provided electronic consent to have their data used for research purposes. All participants in the study downloaded the free application and purchased the associated wireless scale as part of a commercial weight loss program presumably to engage in weight loss; however, motivation was not assessed. This analysis included adult MetaWell users in China that were at least normal weight defined as a BMI of 18.5 kg/m^2^ from October 27, 2016, to December 31, 2017. Subjects also needed to have met a minimum of recorded participation including a baseline weight and a weight at 35 days or beyond. The study design, strategy to analyze the data, actual data analysis, and the writing of the manuscript occurred without company input.

The program directs users to start an initial six-week weight loss program; however, it was commonly continued for longer durations. All users who had a baseline weight and weight recorded around specific time points, 14-day intervals centered on 42, 60, 90, and 120 days, were included in the primary analysis, while all users with a baseline weight and a weight at any time point beyond 35 days were included in the secondary analysis. The sets of users in each time window were not identical. A secondary analysis was performed to investigate average weight at last follow-up that included all individuals with a weight recorded at 35 days or longer. Given that the application is designed to facilitate weight loss, subjects with BMI observations categorized as underweight (BMI ≤ 18.5 kg/m^2^) were excluded. Exceptionally high BMI (≥80 kg/m^2^) observations were also excluded presumed to represent erroneous measurements. To improve the reliability of the analysis and remove outliers, the top and bottom 1% in regard to weight at baseline were also removed from the analysis. Users with a stated age <18 or >100 years were also excluded. Because this cohort was largely based in China, all analysis performed relied on the BMI classification in adult Asians by the World Health Organization (low: <18.5; normal: 18.5–23.0; preobese: 23.0–27.5; obese: >27.5) [14]. Subgroups were evaluated based on age, gender, baseline weight status, and frequency of use. The frequency of use was defined by the number of weights recorded for each person during each time interval studied divided by the number of days a person was in that time period. Tertiles of these frequencies were then made within the time period to create high (highest 1/3 of users), medium (middle 1/3 of users), and low (lowest 1/3 of users) frequency of use categories for comparison. Among participants who were overweight or obese at baseline, we calculated percent excess body weight and percent excess body weight loss according to published recommendations [15].

### 2.3. Statistical Analysis

Means and standard deviations were used to describe continuous variables; counts and percentages were used to summarize categorical variables, both across strata and within stratum. Weight at baseline was determined by taking the median weight within a 3-day period of first user observation. Weights at the end of time intervals were constructed in a similar fashion, but extending the end window to 7 (or 14 in the case of the 120 strata) days before or after the end-date mark. Excess weight was defined as any weight that exceeded that maximum healthy weight as determined by a BMI of 23. Chi-square tests were utilized to investigate if there was an average shift in weights from time 1 to time 2 within stratum, and then further to probe for differences between men and women, age categories, overweight categories, and low-, medium-, or high-frequency weight recorders.

## 3. Results

Overall 251,718 unique individuals, 45% of the original sample were included (measurement at baseline and a minimum of 35 days), of whom 78.6% were female. The mean age was 37.3 ± (9.9), and 73.4% of the population studied was overweight or obese, based on BMI at baseline. 232,759 unique individuals were included in the interval analysis (42-, 60-, 90-, or 120-day interval groups), 78.8% female with a mean age of 37.4 ± (9.9). Baseline demographics are shown in Table 1 for individuals included who had met inclusion criteria and who had recorded participation that spanned at least 35 days.

Overall, users had a mean weight loss of 4.3 kg (CI: ±0.02) and mean follow-up of 120 days (SD: 76.8 days). In the 42-day period, 192,405 individuals were included (Table 2). Mean weight loss for the population was 4.1 kg (95% CI: 0.02) with 58.2% losing >5% of their baseline weight. Men on average lost a mean of 5.2 kg (CI: ±0.05), which was significantly more than in women, who lost a mean of 3.8 kg (CI: ± 0.02, *P* < 0.001). All age groups lost weight at 42 days, with those <30 years of age losing a clinically small, but statistically significant, greater amount than the other age groups studied (*P* < 0.0001). At every interval studied, men lost significantly more weight than women (Table 2, *P* < 0.001 for all comparisons). Although absolute weight loss was greater in men, the weight loss relative to their baseline weight or excess weight was greater in women than in men. However, in all tested intervals of 60 days or more, a greater proportion of women than men achieved over 5% weight loss (*P* < 0.0001 for all comparisons). All age groups lost weight at 42 days, but those <30 years of age had a slightly higher but statistically significant weight loss, when compared to the other age groups (*P* < 0.0001).

**Figure 3:**
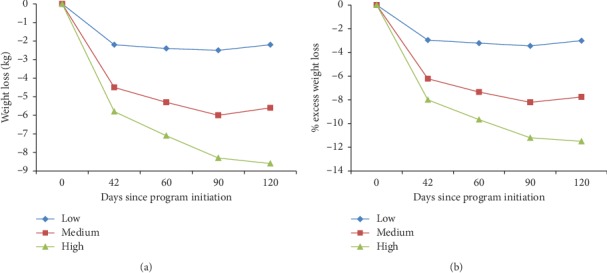
Weight loss results for application users by the frequency of application; tertiles of these frequencies were then made within the time period to create high (highest 1/3 of users), medium (middle 1/3 of users), and low (lowest 1/3 of users) frequency of use categories for comparison. Significant differences were found among all groups at all intervals shown (*P* < 0.001).

High-frequency users recorded an average of 1.2 (SD: 0.41) weight measurements per person day, significantly more than medium (0.76 weights/person day, SD: 0.12) and low (0.33 weights/person day, SD: 0.16) at the 42-day interval (*P* < 0.001 for all comparisons). Weight loss by the frequency of weight measurement at each time interval is shown in Figure 3. High-frequency users had greater weight loss than medium-frequency users, who had greater weight loss than low-frequency users at each time interval studied (*P* < 0.001 for all comparisons).

Among users who recorded at least one weight measurement at 35 days or beyond, the mean duration of app use was 120 days (CI: ±0.3), median 97 days. Women tended to participate longer (mean: 121 days; CI: ±0.34) than men (mean: 115 days; CI: ±0.64; *P* < 0.001). Increased frequency was also associated with longer duration of use. At 120 days, 62.7% of participants had lost at least 5% of their initial weight.

Table 3 displays the weight loss and BMI changes among the 172,464 individuals classified as being either overweight or obese at baseline based on a BMI of ≥23.0 kg/m^2^ (Table 3). Among these, all BMI groups lost significant weight at each interval studied. In general, the groups with the highest excess weight at baseline lost more weight through the intervals studied and similarly had a greater excess weight loss described as a percentage. Of the participants classified as obese at baseline (38,025), 53% moved into the overweight or preobese category by the last observed weight, with 2% moving into a normal BMI category. Of participants categorized as overweight at baseline (146, 646), 30% achieved a normal BMI categorization by the last observed weight.

## 4. Discussion

In this large observational study of greater than 250,000 individuals, people following a commercially available weight loss program using a digital health platform without face-to-face interaction and a simple meal supplement achieved clinically significant short- and midterm weight loss. Individuals defined as obese at baseline, thus most likely to benefit from weight loss, [16] experienced greater weight loss. The scale and duration of this study contribute to the growing evidence base that mobile applications may be a useful tool in combating obesity at a population level with the distinct advantage of being widely accessible, relatively low cost, and without the constraint of face-to-face interaction [5].

This is the largest observational study of a mobile application for weight loss with the largest similar study reporting on approximately 36,000 individuals [17]. Our study differs significantly by adding the hybrid approach use of a wireless home scale and specific nutrition plan. Similar to several randomized trials and other observational studies, this study shows an association between weight loss and application use [18]. It is encouraging that significant weight loss was seen in both genders and all age groups studied, suggesting wide applicability. It is also notable that the program did not involve any specific activity recommendations. This highlights the possible potential in groups with limited mobility. It also allows a possible opportunity for continued evolution and improvement in the program.

The magnitude of the weight loss is highly significant, even in the setting of observational research, with 62.7% of the 120-day cohort losing >5% of their body weight and an average weight loss of 5.4 kg at 120 days. A systematic review of randomized control trials using in-person interventions for weight loss including diet, exercise, and meal replacements showed comparable weight loss of 5 to 8.5 kg at 6 months [19]. Our study shows comparable rates of success but using fewer resources, as our intervention did not require face-to-face interactions or visits to a medical or nutrition facility. Similarly, a recent study showed that an intensive, multifaceted online diabetes prevention program had higher participation but similar weight loss compared to in-person programs [20]. The use of an objective wireless home scale throughout the study, as opposed to self-reporting, adds merit to the weight loss findings [21]. In comparison, the largest available observational mobile app trial noted 31.2% of users losing >5% of baseline weight at 6 months [17], and another large study utilizing a mobile app and scale noted 28.6% of users with >5% at 4 months [22]. The reason for these differences is not clear, but possible factors may include the individualized nutritional plan and nutritional supplement, application design, or increased participant motivation due to financial investment in the program.

Adherence to weight loss interventions has been noted as an important predictor of success [12, 23], a finding which seems to hold true in mobile applications [24]. Our study confirms this finding by showing that users weighing themselves more frequently achieved greater weight loss in a large population, consistent with previous reports [25, 26]. The duration of this study limits insights into weight change beyond 90 days; however, from the available results, there does appear to be a possible plateau in weight loss during the later stages of the study. Possible mechanisms for weight loss plateau include reduced motivation after initial weight loss, difficulty maintaining the recommended diet for extended periods, or achievement of personal goal weight. Further study evaluating the relationship of weight recording and continued weight loss or weight loss maintenance at extended intervals is warranted.

Obesity is a worldwide epidemic with extensive morbidity and mortality initially centered in developed countries; however, it is accelerating at an alarming rate through the developing world [27]. In addition to the public policy and traditional healthcare mechanisms, new tools will be needed to combat obesity at large scale in relatively low-income populations. Digital health programs offer a promising addition to this toolset by leveraging the rise in smartphone connectivity to create scalable solutions to help curb the rising obesity epidemic [6].

### 4.1. Limitations

This study is limited by its observational design which does not allow for a casual association between application use and weight loss. The lack of comparison to a control group is a major limitation because we cannot rule out that motivated people would be able to lose weight on their own while monitoring their body weight regularly. However, epidemiologic and trial data have historically shown that people would rarely lose weight or maintain some weight off without the help of a weight loss program. A randomized control trial would add significantly as would complete follow-up data on all participants. Multiple factors contribute to weight loss [28], but during this study only application usage was evaluated. Further investigation into diet, physical activity, and motivation would be useful additions to future studies. Lack of detailed information regarding the use of the meal supplements limits our ability to analyze the relationship between the use of those supplements and successful weight loss. Extended duration of follow-up would allow for further investigation into the possible prolonged benefits of application use. Information gathered for the study is inherently user generated via the app and thus is likely prone to some error. Failure to self-report weight information is likely confounded with weight loss and is a potential source of bias. Efforts were made to remove seemingly physiologically implausible information while retaining as many users as was feasible. The association between usage of the app and weight loss could be explained by inherent features of people likely to use the app more often (being more motivated, disciplined, or engaged). The study population is based in China and likely composed largely of those of Asian descent; further investigation into other ethnic populations should be considered in future research.

## 5. Conclusion

This study shows clinically significant weight loss among a large Chinese population using a mobile application and wireless scale as a part of a commercially available weight loss program. Greater weight loss was seen in users who weighed more frequently and had elevated baseline BMI.

## Figures and Tables

**Table 1:** Baseline characteristics of the application users.

*N*	251,718
Age (years) (SD)	37.3 (9.86)
Female (% of total)	197,854 (78.6%)
Baseline weight (kg) (SD)	69.2 (14.5)
Baseline BMI (kg/m^2^) (SD)	25.9 (4.19)
Classification (BMI, kg/m^2^)	*N* (% of total)
Normal [18.5∼23)	67,047 (26.6%)
Overweight [23∼25)	53,205 (21.1%)
Preobese [25∼30)	93,441 (37.1%)
Obesity class I [30∼35)	29,750 (12%)
Obesity class II [35∼40)	6,575 (2.61%)
Obesity class III [>40)	1,700 (0.675%)

**Table 2:** Weight loss results at specified time intervals by total groups and demographic subgroups.

	Total	Men	Women	Age <30	Age 30–50	Age >50
Weight loss (kg) (±MOE)						
42 days	−4.1 (0.02)	−5.2 (0.05)	−3.8 (0.02)	−4.3 (0.04)	−4.1 (0.02)	−4.1 (0.05)
60 days	−4.9 (0.02)	−6.2 (0.07)	−4.6 (0.02)	−5.1 (0.06)	−4.9 (0.03)	−4.8 (0.06)
90 days	−5.6 (0.03)	−6.9 (0.10)	−5.3 (0.03)	−5.9 (0.09)	−5.5 (0.04)	−5.4 (0.09)
120 days	−5.4 (0.04)	−6.5 (0.11)	−5.2 (0.04)	−5.6 (0.10)	−5.4 (0.05)	−5.4 (0.10)

>5% total body loss, *N* (% of total)						
42 days	112075 (58.2%)	23432 (57.9%)	88643 (58.3%)	24657 (58.3%)	74128 (58.3%)	13290 (58.1%)
60 days	93047 (64.1%)	18594 (62.8%)	74453 (64.4%)	19132 (63%)	62570 (64.4%)	11345 (64.4%)
90 days	61862 (65.9%)	11604 (63.1%)	50258 (66.5%)	11781 (63.4%)	42320 (66.3%)	7761 (67.4%)
120 days	52800 (62.7%)	9623 (59%)	43177 (63.6%)	10092 (59.7%)	36195 (63%)	6513 (65.7%)

BMI loss (kg/m^2^) (±MOE)						
42 days	−1.5 (0.01)	−1.8 (0.02)	−1.5 (0.01)	−1.6 (0.01)	−1.5 (0.01)	−1.5 (0.02)
60 days	−1.8 (0.01)	−2.1 (0.02)	−1.8 (0.01)	−1.9 (0.02)	−1.8 (0.01)	−1.8 (0.02)
90 days	−2.1 (0.01)	−2.3 (0.03)	−2.0 (0.01)	−2.2 (0.03)	−2.1 (0.01)	−2.1 (0.03)
120 days	−2.0 (0.01)	−2.2 (0.04)	−2.0 (0.02)	−2.1 (0.04)	−2.0 (0.02)	−2.1 (0.04)

**Table 3:** Weight loss results for users with BMI ≥ 23.0 kg/m^2^.

	Overweight (23∼25)	Preobese (25∼30)	Obesity class I (30∼35)	Obesity class II (35∼40)	Obesity class III (>40)
Baseline					

*N*	49298	87362	27993	6199	1612
BMI (SD)	24 (0.57)	27.1 (1.40)	31.9 (1.37)	36.9 (1.36)	42.7 (2.33)
Weight (kg) (SD)	62.9 (5.24)	72.9 (8.16)	88.3 (9.88)	104 (11.8)	122 (14.4)
%EW (SD)	4.47 (2.31)	15.4 (4.32)	28.1 (3.03)	37.8 (2.25)	46.2 (2.78)

42 days					
Weight loss (kg) (±CI)	−3.3 (0.03)	−4.8 (0.02)	−6.6 (0.05)	−7.9 (0.14)	−8.9 (0.30)
%EWL (±CI)	5.37 (0.05)	6.64 (0.05)	7.46 (0.09)	7.6 (0.20)	7.35 (0.38)
BMI loss (kg/m^2^) (±CI)	−1.3 (0.01)	−1.8 (0.01)	−2.4 (0.02)	−2.8 (0.05)	−3.2 (0.12)

60 days					
Weight loss (kg) (±CI)	−3.9 (0.04)	−5.8 (0.03)	−8.1 (0.07)	−9.9 (0.18)	−11 (0.40)
%EWL (±CI)	6.25 (0.06)	7.94 (0.04)	9.22 (0.08)	9.5 (0.17)	9.41 (0.35)
BMI loss (kg/m^2^) (±CI)	−1.5 (0.01)	−2.2 (0.01)	−3.0 (0.02)	−3.5 (0.06)	−4.1 (0.15)

90 days					
Weight loss (kg) (±CI)	−4.2 (0.05)	−6.6 (0.05)	−9.8 (0.27)	−12 (0.27)	−15 (0.63)
%EWL (±CI)	6.7 (0.08)	9.09 (0.06)	11.1 (0.12)	11.8 (0.26)	12.3 (0.53)
BMI loss (kg/m^2^) (±CI)	−1.6 (0.02)	−2.5 (0.02)	−3.6 (0.04)	−4.4 (0.09)	−5.4 (0.24)

120 days					
Weight loss (kg) (±CI)	−4 (0.06)	−6.6 (0.05)	−10 (0.13)	−13 (0.37)	−16 (0.82)
%EWL (±CI)	6.46 (0.09)	9.13 (0.07)	11.6 (0.14)	13 (0.35)	13.3 (0.68)
BMI loss (kg/m^2^) (±CI)	−1.6 (0.02)	−2.5 (0.02)	−3.7 (0.05)	−4.8 (0.13)	−5.7 (0.30)

## Data Availability

The weight loss data used to support the findings of this study are available from the corresponding author upon request.

## Additional Points

*Question*. Is a remote weight loss solution combining a mobile application, wireless scale, and nutritional program with meal replacements effective in a large cohort? *Findings*. Participants remaining in the program lost on average 4.1 kg of weight at 42 days and 5.4 kg at 120 days. *Meaning*. A remote weight loss solution may provide a viable weight loss solution at scale.
